# Notes and Comments

**Published:** 1923-01

**Authors:** 


### HOUSING AND UNEMPLOYMENT.

WE have referred in the foregoing article to the housing question as the most pressing of the health problems of the day. There is possibly no fact with regard to the statistics of unemployment more disturbing to the average citizen than the statement that more than 100,000 of the persons drawing unemployment benefit belong to the building industry. There is not a single member of any council in the kingdom who should remain quiescent while the housing problem for his area remains unsettled. Are the Local Authorities probing this question " every day and in every way," or are they merely wringing their hands and complaining of the iniquity of the Government ? If public opinion in an area is apathetic or ill-informed would it not be well to call public meetings and hammer at the question until the rate-payers themselves demand that the problems shall be settled ? Are the builders being called together in conference with a view to getting at the bottom of the difficulty ? Is it that land is too costly of acquisition ? If so, powers are at hand for compulsory acquisition at a reasonable price. Or is it that the bricklayers are still disinclined to lay the proper number of bricks per day ? If so, let it be cried from the house-tops until for very shame the matter is remedied. Or is it that the private builder or the building societies lack the ready capital to embark upon building enterprise ? Then surely the best interests of the area demand a resort to a system of loans from the State or the Local Authority. Or is it, finally, that the builders are not sufficiently assured in advance of an economic rent ? Then do not the exceptional times justify the Local Authority in giving guarantees subject to one or two essential conditions ? We confess to a certain impatience in this matter, but from every area comes the same distressing tale of home conditions of a certain section of the community which are an abomination.

### THE DEMORALISATION OF THE DOLE.

WE gladly respond to the request of the People's League of Health to support their urgent representations to the late Minister of Health as to the grave effect on the health and the morale of men receiving over long periods payments towards their maintenance in idleness. We do not believe that the large majority of those in receipt of unemployment benefit or Poor Law relief would not be all the better for some part-time employment ; we say part-time advisedly, because we are well aware of the impracticability of giving men whole-time work at an uneconomic wage on works designed primarily for relief. But work on alternate days or for three half-days a week is better than that " unfilled and objectless time " referred to in one of the letters laid before the Minister by the deputation from the League. Dr. Bond referred to the efforts made at Leicester to deal with this question. There they are engaged with good results on big schemes involving the levelling of playing-fields, the laying out of tennis courts, the widening of roads, the white-washing of cattle markets, and so on ; and he expressed the hope that every Council should be stimulated to put forth every possible effort in this direction.

We note with interest that the Government are going forward with the building of two large battleships. We can supply Sir Percy Scott with one reply to his reiterated question, " What is the use of the large battleship ? " One use, at any rate, is to divert into a channel of useful employment a sum of many millions which would otherwise be spent in maintaining men in unwilling idleness. The uses of the battleships when built we leave to the experts, but the building of them is a useful contribution to this question of keeping up the morale and the health of the workers, as to which we are very much concerned.

### THE PRICE OF FOG.

THE medical correspondent of the " Times " recently drew attention to the fact that the price of the fogs which Londoners had been experiencing had been duly exacted. London, as a rule, he said, compares favourably in respect of its death-rate with the rest of the country. In the week ending November 25 it compared most unfavourably. The death-rate for the whole of England and Wales was 12·6 per 1,000, and that for London 14·3 per 1,000. Looking for the reason for this, he finds that the deaths in London from bronchitis and broncho-pneumonia rose from 182 in the previous week to 238.

In the foggy period November 12 to 25 the London death-rate rose sharply above that of the country in general, and this rise corresponded to the increase in the number of cases of bronchitis and broncho-pneumonia. There was no influenza epidemic, and consequently this rise may fairly be ascribed to weather conditions—to the darkness and irritation of the London fogs.

Here surely, says the " Times " correspondent, is a strong argument in favour of smoke abatement. The broncho-pneumonia cases were largely among children, the bronchitis cases largely among old people. Old and young, therefore, are being sacrificed to our criminal carelessness in this matter. A single day of fog in London is more dangerous as a " baby killer " than many air-raids.

### SMOKE ABATEMENT—GERMANY LEADS THE WAY.

LORD NEWTON may be relied upon not to relax his efforts to get a Smoke Abatement Bill—and a more comprehensive effort than that which received a second reading in the House of Lords in July last—passed into law at the earliest possible date. In the course of his speech on that occasion he said that the favourable conditions of industrial Germany, as compared with industrial England, might be attributed, roughly speaking, to three or four reasons—to the almost complete absence of anything in the nature of domestic smoke ; to the more general use of gas for power and so forth ; to the much greater efficiency in the use of fuel, and, lastly, to the greater foresight shown with reference to the expansion of towns and their industries. The contrast between great German industrial towns such as Cologne and Dusseldorf when compared with similar towns such as Manchester and Leeds, in this country, struck him as extremely painful to an Englishman; and the comparison was equally painful lower down in the scale. If you compared, for instance, a town like Essen, perhaps the most smoky town in Germany, with Sheffield, which is the analogous town over here, the comparison was very greatly in favour of Essen. One simple fact illustrates strikingly the superiority of the conditions in Germany, and that is that, whereas in this country in a town like Manchester or Sheffield a manufacturer, as soon as he can get sufficient money, hastens to get as far as he can away from his place of business, in Germany, in a town like Dusseldorf or Cologne, the manufacturer remains and lives in the town because it is an agreeable place of residence.

Unfortunately, said Lord Newton, many of the residents in the industrial districts have become so accustomed to the state of things in which they were born and have grown up, that they are accepting that state of things without making any effort to remedy it. They find their local authorities apathetic, the local authorities find the central authority in London apathetic. Therefore, a large number of persons have come to the unfortunate conclusion that there is nothing to be done and that nothing will be done.

### DIRT AS AN INDEX OF PROSPERITY.

LORD NEWTON finds the belief to be general especially amongst uninstructed people—and it is largely encouraged by a certain class of manufacturers—that dirt means wealth and that the dirtier a country is the more prosperous it is and the happier is the community. This extraordinary delusion appears to have prevailed in very high quarters. About thirty or forty years ago a new railway was opened from a town called St. Helens in Lancashire to some other town, and the Lord Derby of the day, uncle of the present Lord Derby, was invited to perform the ceremony of cutting the sod. The local joke was that it would be impossible to find a sod in St. Helens because, as anybody who knows that town is aware, owing to the industries which were being carried on there with great success, all vegetation had been killed. The day arrived, and Lord Derby came to take his part in the ceremony. All the principal persons were present, and an imported sod was produced which probably had been removed from somebody's lawn tennis ground. Lord Derby divested himself of his coat, was presented with a silver-gilt spade, or whatever the proper implement was, and proceeded to cut the imported sod. Then, turning to his audience, consisting of all the representative persons in the town, he said: "Before I came here I was told that my work would be useless, because there would be no sod in St. Helens to cut. My reply was: 'All honour to St. Helens.'" This sentiment was received with perfect rapture by his audience.

Lord Newton believes that so far from any member of any Government being found nowadays to congratulate a municipality on having successfully destroyed the natural amenities of their neighbourhood they will do their best to remedy such a state of things. We wish him success in his efforts to get the widest possible measure of smoke control which will not prejudice the industry of this country—a limiting consideration to which Lord Newton is fully alive.

### THE HOUSE-FLY.

THE Medical Officer at Portsmouth issued a very interesting little pamphlet on the dangers arising from the house-fly, in which he says that the great danger from flies is that they convey filth and disease germs to food. The fly seen on the dining-table has very likely come straight from the dust-bin, possibly from the expectoration of a consumptive, almost certainly from some form of filth. The house-fly is a disease and filth carrier.

After drawing attention to the diseases which the house-fly may convey, he lays great stress upon the fact that the fly must be tackled in his breeding place. The one essential measure to be put into practice is that no house refuse, kitchen waste, garbage, or stable manure must be allowed to remain long enough for flies to be able to breed out on them.

The last paragraph in Dr. Fraser's pamphlet is worth quoting in full, as it introduces a lightness of touch which is rather welcome in pamphlets of this character, and serves to drive the lesson home. He says:—"It has been stated that in a certain lunatic asylum one of the tests applied to find out if a patient is sufficiently recovered to be discharged is to give him a broom and put him in a room with a water tap turned full on. If he proceeds placidly to sweep up the water without turning off the tap, his standard of intelligence is not deemed to be high enough. The individual who endeavours to get rid of the fly danger by killing individual flies, and at the same time allows their breeding places to remain unheeded is intellectually not far removed."

### MILK WITH A SPECIAL LABEL.

THE Minister of Health has just issued an Order, the Milk (Special Designations) Order, 1922, which provides for the issue of licences:—

(1) By County and County Borough Councils (and in certain circumstances by Urban and Rural District Councils) to producers of "Grade A" milk, and (2) by Sanitary Authorities to (*a*) distributors of "Certified" milk; (*b*) distributors of "Grade A" milk, including "Grade A (Tuberculin tested)" and "Grade A (Pasteurised)"; (*c*) distributors of "Pasteurised" milk.

A farmer applying for a licence to sell "Grade A" milk must submit his herd for examination by a veterinary surgeon nominated by the County Council, who are also advised by the Ministry to satisfy themselves by bacteriological tests that it will be "reasonably probable" that the milk will pass the prescribed tests at the time of distribution.

The sanitary authority must, except in the case of Pasteurised milk, satisfy themselves before granting a distributor's licence that the corresponding licence has been granted to the producer. The Department point out that a distributor who cannot deliver milk that regularly satisfies the prescribed tests is not entitled to hold a licence, even though the failure may be due to the conditions of his source of supply, and not to any fault of his own.

The Order lays down the general conditions governing the sale of milk as "Pasteurised," the essential condition in the matter of treatment being that the milk must be held at a temperature between 145°F. and 150°F. for a period of at least half an hour.

The Minister, we gather, deprecates prosecutions under Section 3 of the Act if a licensee uses the wrong label or otherwise contravenes the conditions of his licence, the appropriate remedy being in that case the revocation of the licence ; but he considers the section "appropriate" for proceedings where a label is used without a licence.

Labels and licences are an earnest of an intention to improve the supply of milk. But we look forward to the time when no milk shall be sold unless it comes from clean cows, milked with clean hands in clean surroundings. The public should be satisfied with nothing less, and the only label that will then be required will be "Milk." There is an unpleasant ring about a sentence in the circular issued to the Councils, in which it is stated that the Minister is advised that the conditions now prescribed for "Grade A" milk are such as should provide milk that is superior to the ordinary milk of the country and reasonably safe under all ordinary circumstances, and at the same time bring the production and distribution of the milk within the competence of all careful dairymen. From which it is to be inferred that "ordinary milk," which is not reasonably safe, will still be finding its way in large quantities into the system of the "ordinary" citizen.

### A DISTURBING MILK CIRCULAR.

THE British Medical Association has added its voice to the protests against Circular 325 issued by the Minister of Health in July last. The Minister expressed the opinion that it was extremely undesirable that a prosecution should be based upon the results of an isolated test when other tests of the particular milk supply had proved satisfactory. So far, this seems unobjectionable. But he went on to suggest that in such cases prosecutions should be instituted only where a series of tests have shown repeated default. And that addition seems to us unfortunate.

### PHYSICAL TRAINING IN SCHOOLS.

THE Board of Education, in a recent circular, again draw the attention of Local Education Authorities to the value of an efficient system of physical training in Public Elementary Schools. Such a system, the Board rightly point out, is a potent auxiliary in the prevention of debility and disease amongst school children, and is relatively inexpensive to maintain. The small expenditure involved, even in a large area, forms but a fraction of the cost which most authorities willingly pay for their school medical service and the care of defective children, and it is reasonable to anticipate that if it is properly developed it will gradually afford relief from some of the heavy expenditure on other "special services." It is because experience has shown the real economy of prevention as compared with the expense of cure that the Board have asked authorities to explore every other avenue of economy so as to be able, even in existing circumstances, to retain or appoint organisers of physical training.

Physical training has also a direct and extremely important part to play in the general education of the child ; it is conducive to discipline, self-control and concentration, a sense of order and responsibility, co-operation with others and generally to the formation of good habits.

In 1917, when the Board were first able to offer grants-in-aid of the salaries of organisers of physical training, they took up two fundamental positions. The first was that if all children in Public Elementary Schools were to receive systematic and regular training in physical exercises, such training must as a rule be carried out by the school teachers. The second was that, in order that the school teachers might keep themselves at a high level of efficiency in this subject, they should have from time to time the special and individual assistance and guidance of experts. The work of the last few years, the Board say, has shown the soundness of this position. Generally speaking, school teachers have proved their competence to take their classes in physical exercises, and have shown that it is not necessary to employ specialist instructors in order to do justice to the children. On the other hand, it is equally clear that even the best and keenest teachers have derived great advantage and encouragement both from the advice and demonstrations given by expert organisers in their schools, and from the classes held for teachers by such experts.

The primary duty of an organiser is to secure a good standard of work in the schools of the area. He has to develop and co-ordinate the physical training in all types of schools, so that all the children in the area may have the opportunities that can only be fully supplied by well-planned, continuous and progressive training. He can also render very valuable service by interesting himself in and stimulating the development of all analogous forms of physical recreation, as, for example, by promoting the formation of schools' athletic associations, by the linking-up of voluntary juvenile organisations, and by encouraging, possibly through voluntary agencies, such activities as camping, swimming and organised games. All this requires considerable time and no slight powers of organisation ; it postulates also the possession of character, leadership and imagination.

The Board, therefore, would appear to deprecate any loose arrangements with regard to the organisation of physical training, whether by the appointment of peripatetic specialist teachers of physical exercises, or the detaching of ordinary teachers from their ordinary class-work to visit other schools, and they intimate that in general they are not prepared to recognise for grant expenditure on the employment of persons who are not recognised organisers.

### LIVERPOOL'S SCHOOL OF HYGIENE.

IN the excellent Report made by the Medical Officer of Health for the City of Liverpool for the year 1921 there is an interesting reference to the work of the School of Hygiene. It is pointed out that the need for an educated public opinion in regard to health matters was realised nearly forty years ago in Liverpool, and also that Liverpool presented a field without an equal in this country for the application of sanitary measures. The teaching of the subject of Public Health was introduced at the Medical School in 1886. The interest increased as the School itself increased, and a few years after the establishment of the University of Liverpool an important section, known as the School of Hygiene was devoted to the teaching of this subject, and an unendowed Professorial Chair of Public Health was established. An Exhibition designed with the object of assisting the teaching of Public Health to every person interested, whether the medical man, the medical officer of health, the health inspector or visitor, midwife or school teacher, as well as the general public, was formed. When the Civic and University conditions permitted, a highly important combination was effected by which the whole of the technical investigation in the interests of the Public Health carried on by the City Bacteriologist, and the City Analyst, as well as by the teachers of Hygiene, were incorporated in the one building, specially erected for the purpose by joint funds provided by the two bodies mentioned, and, as far as the specific object of teaching is concerned, supplemented by generous benefactions. The University itself gave most sympathetic encouragement to this latter aspect and further recognised its importance by conferring not only a Diploma but a Degree also in the subject of Public Health.

The work of this School has proved conspicuously successful; the Inspector appointed last year by the General Medical Council to attend and report upon the Examinations at the various Universities stated that the opportunities offered at Liverpool for practical instruction in public health and its administration were exceptionally good.

### THE NEW DENTISTS' ACT.

THERE have been added to the Roll of Dentists by the Act 8,000 new names. Such a dilution by the untrained of the comparatively small body of trained dentists would be a serious matter at any time. At the present time the almost universal presence of mouth sepsis with its concomitant general symptoms constitutes a national threat than which there is no greater. It becomes of increasing importance that the public should choose its dental attendant with the greatest care. There are the trained and the untrained, the qualified and the unqualified. No Act of Parliament alone can transform the one into the other. There never was a greater need for scientific dentists. The mouth is beyond doubt the focus from which almost any disease may arise. The press, both scientific and public, have been calling attention to the subject. The original Dentists' Act of 34 years ago, had it been drawn up properly, or had the decision of the Lords of 1909 been different, would have before now provided the nation with a really useful and sufficiently large body of dental practitioners. As it is, the absence of any real scientific training, and perhaps the presence of a commercial instinct, may tend in one direction to wholesale and unnecessary extraction of teeth, with consequent wholesale manufacturing of plates, and in the other the preservation of teeth and roots which constitute a menace to health.

### SMALLPOX DECLINES.

FORTUNATELY the outbreak of smallpox, which threatened serious developments during the past month, has now definitely declined. This alone is a matter for satisfaction, but there are two or three other points which should be appreciated.

In the first place, the modern methods of control have again proved efficient, namely, constant search for and vaccination of all possible contacts, a surveillance of these until the period of incubation is past, and immediate hospital isolation of every case the moment it is discovered.

Also the direct effect has been to raise in a remarkable way the percentage of vaccinated people. It is, for example, noteworthy that in Poplar itself, where vaccination had been notoriously neglected and in whose workhouse the original outbreaks took place, particularly free resort to vaccination has now occurred! The same news comes from most parts of the country, and it was indeed time that some form of public stimulation took place. A stage had been reached when in several areas at least 50 per cent. of the children had never been vaccinated and a totally unprotected population was springing up apace. For that reason this threatened outbreak has served a useful purpose.

One great disadvantage of such haste is, however, the heavy pressure upon the facilities available. Vaccination is a small enough operation, but it demands care and thoroughness. If the public would only take the trouble to be vaccinated and revaccinated in normal times and not allow the process to resemble so closely a panic measure, there would be far fewer "bad arms" and unsatisfactory results, not to mention the ultimate saving of smallpox outbreaks.

### THE STERILISATION OF TUBERCULOUS SPUTUM.

THERE seems to be a general impression that if a strong antiseptic lotion is put into a mug, the sputum coughed into it by the subjects of pulmonary tuberculosis will soon be sterilised. Unfortunately, this is not true, and it seems at first paradoxical that the stronger the solution of certain antiseptics, the less effective is their bactericidal action on the lumpy sputum of consumptives. Such disinfectants as perchloride of mercury coagulate the exterior of these lumps, creating an impermeable capsule within which tubercle bacilli are perfectly safe so long as the lump is not broken up. Experiments with lysoform have also proved this to be worthless as a disinfectant. Antiformin is more effective, as it breaks up and dissolves lumps of sputum, but it has to be heated to a temperature of about 55°C. before it exerts a rapidly disinfectant action on tubercle bacilli. At the last German Tuberculosis Congress, Professor Paul Uhlenhuth claimed to have at last solved the problem of the chemical destruction of tuberculous sputum. It is illustrative of the complexity of this problem that he and his assistants have been engaged on it for the past 15 years, and more than 1,000 guinea pigs have been used, so that the presence or absence of tubercle bacilli in sputum treated by various methods could be demonstrated by inoculation. Professor Uhlenhuth and his assistants seem to have stumbled on a satisfactory disinfectant more or less by accident; in 1919, they found, to their great surprise, that certain kresol substitutes for soap, which contained small quantities of sodium hydroxide but no soap, destroyed tubercle bacilli in sputum. A strong alkali has long been known to break up tuberculous sputum, but even in as strong a solution as 20 per cent., sodium hydroxide does not destroy tubercle bacilli in broken-up sputum. The effectiveness of these kresol compounds, such as K-lysol and beta-lysol, would, therefore, seem to depend on two factors—the mechanical breaking-up of the sputum by the alkali present, and the chemical destruction by the lysol of the tubercle bacilli thus exposed. Professor Uhlenhuth found that tubercle bacilli in sputum were destroyed within four hours when 50 c.c. of sputum were added to 100 c.c. of a 5 per cent. solution of alkali-lysol containing 4 per cent. alkali and 65 per cent. kreosole. Professor Uhlenhuth has done well not only in pointing the way to effective sterilisation of tuberculous sputum, but also in exposing the common fallacy that any disinfectant will do.

### THE FUTURE OF THE POOR LAW HEALTH SERVICES.

We turned with interest to the address of the President at the annual meeting of the Association of Poor Law Unions, to see what he had to say on the question of the future of the Poor Law Health Services. We found it very unprofitable. Our interest in the matter is, of course, mainly, if not wholly, that of the co-ordination of our various Health and Hospital services. The primary object of the Association being to preserve, to defend, and, where necessary, to extend and adapt the present Poor Law System to varying social needs, we can understand the presidential outburst, but frankly it will require something more reasoned and reasonable to persuade public opinion that the arguments as to the extravagance and overlapping of the Poor Law system are all unsound. That the question of the abolition of the guardians "did not come up at the recent General Election," we have no difficulty in agreeing. We find it, therefore, the more difficult to understand why the Maclean Report is as dead as Queen Anne because Mr. Donald Maclean was rejected in Scotland and Dr. Addison failed to secure re-election in London. When in addition we read that the overthrow of the late Government was the "Nation's reply to Lord Ullswater"—with whom the President of the Poor Law Association is very annoyed because his evidence on the Maclean Report was refused by the Royal Commission on London Government—we are still more puzzled. Poor Lord Ullswater has survived his long spell of office in the Speaker's Chair in the House of Commons to learn that he is "unjust, partial, and unjudicial." These expressions and those others at which we have glanced rather detract from the value of the president's opinion that the sooner the Ministry of Health is disestablished the better, nor can they be regarded as a helpful contribution to the question of the future of the Poor Law Health services.

### CO-OPERATION BETWEEN A GENERAL AND SPECIAL HOSPITAL.

We have to record a most interesting innovation in the Hospital world which breathes a spirit of enlightenment and marks a reformation in procedure of an advanced and progressive character. We refer to the scheme of co-operation now adopted by the Middlesex and St. Luke's Hospitals, which is, we believe, the first instance in this country of an alliance between a large General Hospital and a registered hospital for Mental Diseases; and we must congratulate the Boards of these two institutions on the important step which they have now taken.

This step has not been prompted by any recent agitation, for the idea has been thought out and discussed for many years past, but only now brought to a concrete and practical conclusion. Under the scheme all cramping restrictions will be removed and the two hospitals, by their mutual co-operation in respect of funds, material, personnel and experience in management, will attain the ideal in the treatment of early mental and functional nervous disorders. while securing economy with more rapid and efficient treatment of the patients.

Two new wards for male and female In-patients will be established in the Middlesex Hospital where the cases will be treated by members of the Medical Staff of St. Luke's Hospital under the care of its trained nurses. A Special Out-patient Clinic will be established for dealing with border-line cases, and this will practically constitute a Psychiatry Section of the Neurological Department. Thus as far as Out-patients are concerned the scheme involves close co-operation of the two hospitals, and is a step towards a system which can and may be extended with advantage in other directions.

There is a marked disinclination among people suffering from early mental disorders to consult the specialist at a hospital known to deal with only this class of disease. Consequently, such patients are far more likely to attend at a General Hospital for treatment on the appearance of their first symptoms. The establishment of a Department of Psychiatry in charge of specialists at a General Hospital should prove very valuable, for it is universally recognised that early treatment on the right lines is a long step towards effecting a cure; delay is dangerous or even fatal to a successful issue. Further, from the point of view of the patients, the stigma which is popularly connected with a hospital dealing only with mental disease is avoided under this scheme, for the border-line cases, if requiring further attention than can be given in the Out-patient Department will be admitted as In-patients to the new wards at the Middlesex Hospital on the same footing as ordinary patients in the general wards.

An additional benefit accruing to the patients will be that the full resources of a large General Hospital will be at their disposal for physical examination, special methods of treatment, pathological investigations in properly equipped laboratories, and for any medical or surgical treatment required for their relief. In this way certification may be avoided, and prospects of cure improved.

The results of this interesting combination of forces will be watched with great interest, and we are confident that they will fully justify the scheme which has been so cordially arranged between the two hospitals.

### SEAMEN'S HOSPITAL, MALTA, OPENED.

THE King George V. Merchant Seamen's Memorial Hospital at Malta, which was formally opened on St. Andrew's Day, is a fit memorial of the services rendered by the mercantile marine during the war, besides being a valuable addition to the institutions on the island. Thus is realised the scheme launched in 1918 by Lord Methuen, when governor of the island. A sum of £20,000, including interest, was raised by his appeal, and Capt. and Mrs. Wisely, the latter Sir Donald Currie's daughter, have provided an endowment of £25,000. The foundation-stone was laid on March 27, 1919, and construction, because of the difficulties of the site, began in August, 1920. The hospital, of which Mr. Pirie is the architect, and Mr. Robertson chairman of the Building Committee, contains 32 beds, and is capable of extension. The medical and nursing staff have arrived, and it is hoped that the realisation of the original scheme may lead the public to complete it by raising the second half of the endowment fund desired, of which Mrs. Wisely has given the first. It will be recalled that the new institution is not the first Merchant Seamen's hospital in Malta. In 1882, part of the Clapp-Zammit Hospital at St. Julian's was so known. It contained 150 beds, to which admission was given by the payment of sums assessed according to rank. The nursing staff consisted of the Sisters of the Little Company of Mary. One of the points made by Lord Methuen in his appeal was the need for a hospital for merchant sailors and others under English management. The new Memorial Hospital will make the fifth hospital of importance on the island.

### LEPROSY IN THE UNITED STATES.

LEPROSY, says Surgeon-General Cumming, of the U.S. Public Health Service, though comparatively rare in continental United States, is still a public health problem of greater seriousness than seems to be popularly supposed. Leprosy was probably introduced to the new world in the days of the slave trade. Since then it has extended widely among island populations, where semi-civilisation prevailed.

When the Philippines were acquired, several thousand lepers had to be, and still are, segregated in a settlement at Culion. Foci of the disease exist on the Pacific coast, in the North-West, in the Gulf States, and along the Atlantic seaboard.

It was not until 1889 that the U.S. Government took its first steps to prevent lepers from coming to the country; and the preventive laws are now sufficiently explicit. Nevertheless, the long period during which the disease incubates before declaring itself makes it very difficult to shut out all potential lepers. To meet this difficulty the law provides that for three years after admission any alien leper may be deported if the cause of the disease existed before he came to the United States.

In February, 1917, Congress provided for a national home for lepers to be administered by the Public Health Service; but so strongly did the various States object to the founding of such an asylum within their borders that it was not until four years later that the Service was able to carry out the provisions of the Act by acquiring the State leper home in Louisiana.

The gloomy outlook for lepers has recently been tinged with hope by the discovery of a mode of treatment involving the use of chaulmoogra oil, which for 200 years has been reputed to be of beneficial effect for leprosy. The oil itself is absorbed slowly and is apt to cause painful abscesses; but the hypodermic injection of its ethyl esters, devised by Dean, has supplied a way by which it may be used without undue suffering. Since 1912 a total of 183 patients have been paroled from the Kalihi Investigating Station of the Public Health Service in Hawaii (140 of them since July 9, 1919) as being apparently cured. Of those who have received the chaulmoogra oil derivatives only 12 (8 per cent.) have returned for further treatment. While it is too early to say that a specific cure for the disease has been found, it is certain that the ethyl esters constitute a most valuable agent in the treatment of leprosy, especially for young persons and those in the early stages of the disease; in older persons and advanced cases the indications are less promising.

Fortunately for England the leprosy problem is not acute and the powers exercised by port sanitary authorities have so far been found sufficient to prevent any influx of the disease, which by local segregation was stamped out long ago. It is said that the last indigenous leper died in Shetland about 1798. To-day there are not more than 50 sufferers from the disease in the whole of the United Kingdom, and many of these are unfortunates who contracted the malady while on Empire service. All have nowadays sufficient of comfort and care, as instanced by the excellent work of the Homes of St. Giles organised by the Anglican Brothers and Sisters of the Society of Divine Compassion.

An architectural competition for the plans of a small hospital has been announced by the Modern Hospital Publishing Committee, Chicago. The premiums are $500, $300 and $200 for the first three designs, which must be submitted by January 15 to the committee at 22 East Ontario Street, Chicago, U.S.A. Conditions can be seen at the library of the R.I.B.A., 9 Conduit Street, W.1.

